# A Comprehensive Assessment of Nutritional Value, Antioxidant Potential, and Genetic Diversity in *Metapenaeus ensis* from Three Different Populations

**DOI:** 10.3390/biology13100838

**Published:** 2024-10-19

**Authors:** Yundong Li, Juan Chen, Song Jiang, Qibin Yang, Lishi Yang, Jianhua Huang, Jianzhi Shi, Yan Zhang, Zhibin Lu, Falin Zhou

**Affiliations:** 1 South China Sea Fisheries Research Institute, Chinese Academy of Fishery Sciences, Key Laboratory of South China Sea Fishery Resources Exploitation and Utilization, Ministry of Agriculture and Rural Affairs, Guangzhou 510300, China; liyd2019@163.com (Y.L.); chenj29725@163.com (J.C.); tojiangsong@163.com (S.J.); yangqibin1208@163.com (Q.Y.); yangls2016@163.com (L.Y.); huangjianhua@scsfri.ac.cn (J.H.); shijianzhi1989@163.com (J.S.); zhangyan202205@126.com (Y.Z.); lzb9502@163.com (Z.L.); 2 Key Laboratory of Efficient Utilization and Processing of Marine Fishery Resources of Hainan Province, Sanya Tropical Fisheries Research Institute, Sanya 572018, China; 3 Shenzhen Base of South China Sea Fisheries Research Institute, Chinese Academy of Fishery Sciences, Shenzhen 518108, China; 4 College of Fisheries and Life Sciences, Dalian Ocean University, Dalian 116023, China; 5 Guangzhou Nansha Fishery Industry Park Co., Ltd., Guangzhou 510000, China

**Keywords:** *Metapenaeus ensis*, germplasm resources, genetic diversity, population, nutritional components

## Abstract

Due to its high tolerance to salinity and temperature, as well as its strong adaptability, *Metapenaeus ensis* holds an important position in the Chinese aquaculture industry. However, studies on the evaluation of its germplasm resources remain insufficient. This research conducted an in-depth comparative evaluation of *M. ensis* from three representative farming regions in China: Sanya, Zhuhai, and Raoping. The nutritional analysis of muscle tissue showed no statistically significant differences in crude ash, moisture, and crude protein content among the populations (*p* > 0.05). However, significant differences were observed in crude fat and total sugar content (*p* < 0.05). The MeSY and MeRP populations had higher crude fat content than the MeZH population (*p* < 0.05), while the MeZH population exhibited the highest total sugar content. In terms of amino acid composition, the MeSY population had relatively higher total essential amino acid content and proportion, as well as higher total amino acid content, both of which were statistically significant (*p* < 0.05). A fatty acid composition analysis further highlighted the advantages of the MeRP population in several key fatty acids (*p* < 0.05). Physiological and biochemical analyses showed no significant differences among the three populations in total antioxidant capacity, superoxide dismutase activity, or catalase activity (*p* > 0.05). A genetic diversity analysis indicated that *M. ensis* has relatively low diversity, with the MeSY population showing higher SNP density and nucleotide diversity. A genetic differentiation analysis revealed significant genetic differentiation between the MeSY and MeZH populations, while differentiation between the MeZH and MeRP populations was relatively smaller. This comprehensive assessment of nutritional components, amino acids, fatty acids, antioxidant capacity, and genetic diversity highlights the advantages of germplasm resources from different regions. These findings provide valuable insights for future research on the genetic characteristics and breeding potential of *M. ensis*.

## Simple Summary

*Metapenaeus ensis*, valued in Chinese aquaculture for its salinity and temperature tolerance, has seen limited research on germplasm evaluation. This study compared *M. ensis* from Sanya, Zhuhai, and Raoping, China. A nutritional analysis showed no significant differences in ash, moisture, and protein across populations but did reveal variations in fat and sugar content, with Sanya and Raoping having higher fat and Zhuhai having higher sugar. Amino acid composition favored Sanya’s population, while a fatty acid analysis highlighted Raoping’s advantages. Physiological and biochemical characteristics were similar among populations, but genetic diversity was low, with notable differences between Sanya and Zhuhai populations. The study provides insights into regional germplasm advantages and genetic characteristics for future *M. ensis* research and breeding.

## 1. Introduction

*Metapenaeus ensis*, belongs to the order *Decapoda*, the suborder *Natantia*, the family *Penaeidae*, and the genus *Metapenaeus*. Commonly referred to as oilback shrimp, sand shrimp, reed shrimp, etc. [1]. It is a euryhaline shrimp species, native to the Indo-West Pacific region, including the East China Sea and South China Sea of China, Japan, Indonesia, Malaysia, Australia, and other places [1,2]. In China, the cultivation of the *M. ensis* is mainly concentrated along the coasts of the East China Sea and the South China Sea, especially in the estuarine and nearshore waters of the southern coastal areas such as Fujian, Guangdong, Guangxi, and Hainan [2]. They can grow in both saltwater and freshwater, and with their high-temperature tolerance, low-salinity resistance, rich protein content, and uniquely delicious taste, they have won widespread favor in the market [3].

Although research on the nutritional components of shrimp has been quite extensive [4,5,6], studies on the nutritional assessment and genetic resources of the *M. ensis* are relatively limited. Currently, research on the *M. ensis* tends to focus more on its disease resistance [7] and adaptability to environmental changes [8]. Researchers [9] analyzed the muscle nutritional components of wild *M. ensis* of different sizes from the Raoping area in China. The results showed that while the protein content was relatively high, the content of essential amino acids and delicious amino acids was low, especially the content of shrimp-flavored amino acids, which may be related to the long-term accumulation of flavor substances. Although the essential amino acid index was high, the overall composition of amino acids was not balanced. The fat content was at a moderate level. A study on the *M. ensis* examined the growth, antioxidant capacity, digestive enzyme activity, and muscle growth-related gene and protein synthesis-related gene expression in response to different dietary protein levels, concluding that a feed protein content of 38.59% was most beneficial [10]. A study on the genetic diversity of the *M. ensis* populations in the Tam Giang-Cau Hai lagoon in Vietnam, discovered a high degree of genetic diversity by analyzing the mtCOI gene of 91 individuals, identifying 34 unique haplotypes and 38 genetic variations, indicating a high level of gene flow [11]. In addition, in recent years, researchers have assembled the complete mitochondrial genomes of the *Metapenaeus affinis* and the *M. ensis*, providing important information for the study and utilization of shrimp germplasm resources [12].

Although existing research has provided preliminary crucial knowledge for understanding the conservation of the genetic resources and genetic diversity of the *M. ensis*, research in this field is still relatively scarce, especially in China, where in-depth studies on specific geographic areas are particularly lacking. In the face of the growing impact of climate change and human activities on marine ecosystems, regular monitoring and assessment of species that are ecologically and economically vital, such as the *M. ensis*, have become particularly urgent. To address this research gap, this study selected three representative farming areas in China: Sanya, Zhuhai, and Raoping, and conducted a systematic sample collection. We sampled the muscle tissue of the *M. ensis* from these areas, assessing not only their nutritional value but also establishing a comprehensive genetic resource database. This study further utilized molecular genetic methods to assess the genetic diversity of the *M. ensis* populations from these three areas. Through these comprehensive research activities, we hope to provide a solid scientific foundation for the long-term sustainable management and conservation strategies of the *M. ensis*. At the same time, we also hope that the methods and findings of this study can provide a reference for the conservation efforts of other marine species.

## 2. Materials and Methods

### 2.1. Ethical Statement

The animal study was reviewed and approved by the Animal Care and Use Committee of the South China Sea Fisheries Research Institute. All experiments were conducted in full compliance with national laws, regulations, and ethical guidelines regarding animal experiments.

### 2.2. Sample Collection

Following the principles of biodiversity conservation and relevant policies and regulations, we randomly collected population samples of *M. ensis* from three regions: Sanya, Zhuhai, and Raoping. Thirty prawns were collected from each region, totaling 90 prawns. The specific collection details are provided in Table 1, covering one genetic breeding center and two leading enterprises. Samples from the MeSY and MeZH regions were obtained from prawn populations raised under a “pond combined with industrial workshop” farming model, while those from the MeRP region were collected from populations raised under a “cage combined with industrial workshop” farming model. All samples grew in similar farming environments, with the same brand of feed, and were aged between 3 and 4 months. The water temperature was strictly maintained at around 28 °C. Additionally, water quality parameters such as pH, dissolved oxygen, salinity, ammonia, nitrite, and nitrate were measured using a calibrated pH meter, a dissolved oxygen meter, a salinity meter, and chemical test kits with colorimetric methods. The results indicated that the water quality parameters across the different populations were similar, demonstrating that farming conditions were largely consistent across the regions. At each collection site, we precisely measured the body length and body weight of 30 *M. ensis*. Subsequently, we randomly selected 5 live prawns from each site, extracted 1 g of muscle tissue from each prawn, placed it into 2 mL centrifuge tubes, and stored them in a −80 °C freezer for subsequent basic nutrient analysis. The remaining samples were used for further genetic diversity analyses to assess the genetic characteristics of the germplasm resources.

### 2.3. General Nutrition Analysis

In each sampling area, we randomly selected three healthy *M. ensis* in the intermolt stage and collected muscle tissue samples for detailed nutritional analysis. The moisture content was precisely analyzed according to the national food safety standard GB 5009.3–2016 [13]. Protein content was measured following GB 5009.5–2016 [14] to ensure accuracy. Fat content was evaluated based on GB 5009.6–2016 [15]. Additionally, ash content was determined according to GB 5009.4–2016 [16], and the total sugar content was assessed following GB/T 15672–2009 [17]. These standards were rigorously followed to ensure that our analysis was both comprehensive and precise.

### 2.4. Physiological Analysis

In accordance with national food safety standards, we analyzed the hydrolyzed amino acid content in the samples following the guidelines outlined in “Determination of Amino Acids in Food” (GB 5009.124–2016) [18]. Simultaneously, we measured the fatty acid content of the samples according to the standard “Determination of Fatty Acids in Food” (GB 5009.168–2016) [19]. For antioxidant enzyme activity assays, we used the WST-8 kit provided by the Nanjing Jiancheng Bioengineering Institute to measure superoxide dismutase (SOD) activity(A001-1-2) and the total antioxidant capacity (T-AOC) kit(A015-1) to assess total antioxidant capacity. Additionally, we utilized the catalase (CAT) kit(A007-1-1) from Jiancheng Bioengineering to determine CAT activity. Throughout the process, we strictly followed the operational instructions provided with the kits, meticulously prepared the samples, accurately added reagents to initiate the reactions, and terminated the reactions at the prescribed times. We then measured the reaction products, precisely calculated enzyme activity, and thoroughly documented the experimental results.

### 2.5. Genetic Diversity

This study conducted an in-depth genetic diversity analysis on muscle samples from live shrimps randomly selected from various regions. Once the genomic DNA of the samples was tested and confirmed to be of sufficient quality, it was fragmented through ultrasonication, a mechanical process. The fragmented DNA was then purified, followed by end repair, 3′ end A-tailing, and sequencing adapter ligation. Agarose gel electrophoresis was used to select DNA fragments of the desired size. These fragments were then amplified by PCR to create sequencing libraries. The constructed libraries were initially evaluated through quality control checks. Subsequently, high-throughput sequencing was performed on the Illumina platform (Illumina, Inc., San Diego, CA, USA), and the raw data were preprocessed using fastp software (0.23.2) [20] to remove reads with over 50% adapter sequences, bases with a quality score below 12 exceeding 50%, and reads with more than 10% N content, ensuring data cleanliness and quality. The clean data were then efficiently aligned using Sentieon (v201711.03) software [21] combined with the optimized Burrows-Wheeler Aligner (BWA) MEM software (Wellcome Trust Sanger Institute, Cambridge, UK) [22], significantly enhancing alignment speed while maintaining high accuracy [23]. For variant detection, Sentieon (v201711.03) utilized the HaplotypeCaller in joint-calling mode to generate gVCF files and identify SNPs within the population. To ensure the accuracy of SNP detection, stringent quality control was applied using GATK-recommended filtering parameters. High-confidence SNPs with a genotype call rate exceeding 60% were further screened using the SelectVariants and VariantFiltration tools. Finally, SNP detection was performed on BAM files using GATK in combination with VCFtools and localized scripts, calculating population genetic diversity parameters including SNP density, nucleotide diversity, inbreeding coefficient, polymorphic information content, and observed heterozygosity. Additionally, the F_ST_ index was calculated to assess the degree of genetic differentiation between populations. To further improve data quality, low-quality data filtering was also performed using SOAPnuke software (https://github.com/BGI-flexlab/SOAPnuke, 2.X version, accessed by 14 October 2024) [24]. This comprehensive high-throughput sequencing and bioinformatics analysis pipeline provided a solid foundation and valuable information for assessing and understanding the genetic diversity of shrimp samples.

### 2.6. Data Analysis

In this study, we applied SPSS 16.0 software to conduct a detailed one-way analysis of variance (ANOVA) on nutritional components, amino acids, fatty acids, and oxidative stress indicators, using a significance level of *p* < 0.05. If the result was *p* > 0.05, it was considered not statistically significant. Upon detecting significant differences, Duncan’s multiple range test was employed to further explore specific intergroup differences. The experimental data were presented as a mean ± standard deviation, ensuring the accuracy and reliability of the results. In terms of the genetic diversity analysis, we quantitatively assessed genetic variation through a series of calculation formulas, including SNP density, π diversity index, F heterozygosity index, PIC polymorphic information content, observed heterozygosity (Ho), and F_ST_ genetic differentiation index. The specific calculation formulas are as follows: SNP density = SNP number/genome size in Kb, π = n/(n − 1) ∑pipjπij, F_HOM_ = (Ho − He)/(Ho − He), PIC = 1 − ∑pi^2^ − ∑∑2pi^2^pj^2^, Ho = het/(het + hom), F_ST_ = (HT − HS)/HT.

## 3. Results

### 3.1. General Nutrition

In the nutritional analysis of *M. ensis* muscle tissue, we found no significant differences (*p* > 0.05) in the basic nutritional components (crude ash, moisture, and crude protein) among the three farmed populations. However, there were significant differences (*p* < 0.05) in crude fat and total sugar (Table 2). Specifically, the ash content was similar across the three populations, ranging from 1.40 to 1.47 g/100 g, and the moisture content was also comparable, falling within the range of 74.50–75.30 g/100 g. The crude fat content was slightly higher in the MeSY and MeRP populations, at 0.97 g/100 g and 1.03 g/100 g, respectively, while it was slightly lower in the MeZH population, at 0.83 g/100 g. Crude protein content was highest in the MeSY population, reaching 22.10 g/100 g, followed by the MeRP population at 21.17 g/100 g, and the MeZH population at 20.80 g/100 g. The total sugar content was slightly higher in the MeZH population at 0.36%, compared to 0.34% in the MeSY population, and lowest in the MeRP population at 0.29%.

### 3.2. Amino Acids

**Figure 1:**
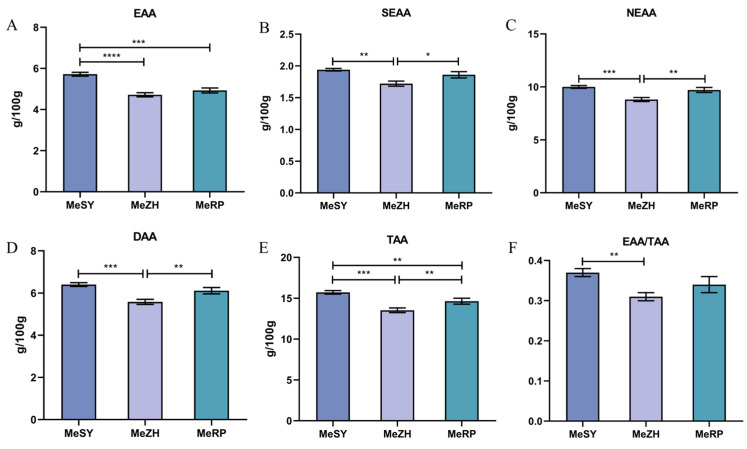
Muscle amino acid composition and content of three groups of *M. ensis*. (**A**): EAA: essential amino acids. (**B**): SEAA: total semi-essential amino acids. (**C**): NEAA: total non-essential amino acids. (**D**): DAA: delicious amino acids. (**E**): TAA: total amino acids. (**F**): EAA/TAA: essential amino acids/total amino acids. Different numbers of symbols between treatments indicate significant differences (* 0.01 < *p* < 0.05; ** *p* < 0.01, *** *p* < 0.001, **** *p* < 0.0001).

A detailed analysis of amino acid content in the MeSY, MeZH, and MeRP populations revealed the presence of 17 amino acids, including 7 essential amino acids (EAAs), 2 semi-essential amino acids (SEAAs), and 8 non-essential amino acids (NEAAs) (Table 3, Figure 1). The MeSY samples showed the highest levels in both the total amount of essential amino acids (EAA) and the ratio of essential amino acids to total amino acids (EAA/TAA), with specific values of 5.72 g/100 g and 0.37 g/100 g, respectively. This indicates a significant advantage of MeSY in providing essential amino acids. The MeSY samples also had relatively high levels of semi-essential amino acids (SEAA) and non-essential amino acids (NEAA), at 1.94 g/100 g and 10.00 g/100 g, respectively. In terms of total amino acid (TAA) content, MeSY ranked first with 15.72 g/100 g. In contrast, the MeZH samples had lower amino acid levels than MeSY in most cases, but higher levels of certain amino acids such as cysteine (Cys), valine (Val), and isoleucine (Ile) compared to MeRP. Additionally, specific amino acids like glutamic acid (Glu), glycine (Gly), and alanine (Ala) were significantly higher in MeSY than in the other two samples. Significant differences (*p* < 0.05) were observed among the three groups for all amino acids except for serine (Ser), methionine (Met), and histidine (His).

### 3.3. Fatty Acids

**Figure 2:**
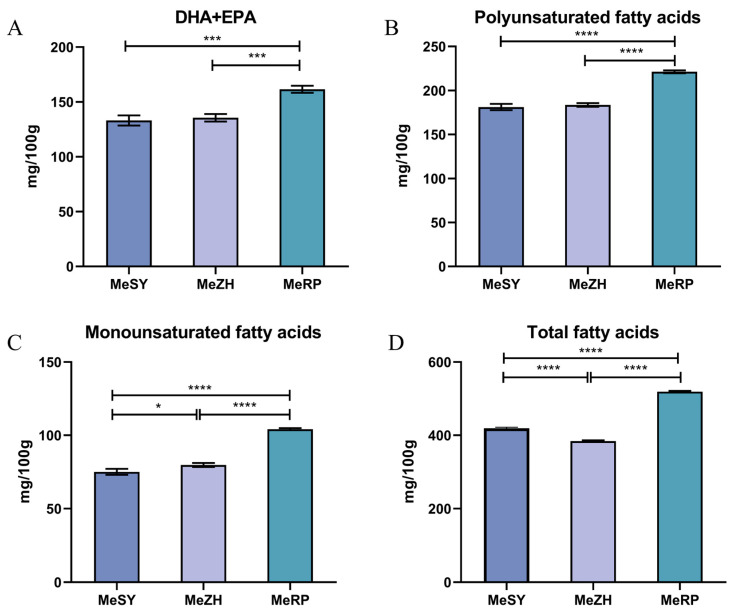
Muscle fatty acid composition and content of three groups of *M. ensis*. (**A**): the content of DHA + EPA. (**B**): the content of polyunsaturated fatty acids. (**C**): the content of monounsaturated fatty acid. (**D**): the content of total fatty acid. Different number of symbols between treatments indicate significant differences (* 0.01 < *p* < 0.05; *** *p* < 0.001, **** *p* < 0.0001).

In the analysis of fatty acid composition across the three different populations (MeSY, MeZH, MeRP), a total of 12 fatty acids were detected, ranging from palmitic acid (C16:0) to DHA (C22:6n3) (Table 4, Figure 2). The experimental results showed significant differences (*p* < 0.05) in all fatty acids across the three samples, except for C20:2. The overall fatty acid content was higher in the MeRP population, with all detected fatty acids, except for C20:2 and C22:1n9, being significantly higher than those in the MeSY and MeZH populations. Although the MeSY and MeZH populations each had advantages in certain specific fatty acids, their overall fatty acid content was similar. Specifically, the total fatty acid content averaged 418.95 mg/100 g in the MeSY samples, 384.35 mg/100 g in the MeZH samples, and the highest at 519.30 mg/100 g in the MeRP samples. The MeRP population also showed significantly higher levels of ΣMUFA, ΣPUFA, and DHA + EPA compared to the MeSY and MeZH populations. Significant differences (*p* < 0.05) were observed among the populations for ΣMUFA, ΣPUFA, DHA + EPA, and total fatty acid content.

### 3.4. Oxidative Stress Indicators

**Figure 3:**
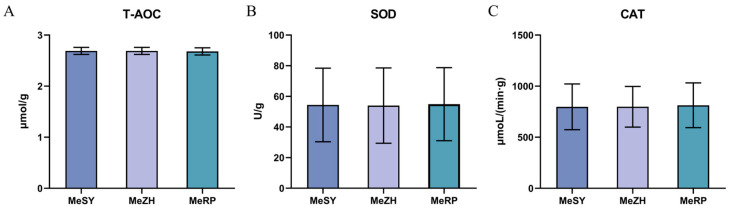
Muscle physiological and biochemical indicators composition and content of three groups of *M. ensis*. (**A**): The activity of T-AOC. (**B**): The activity of SOD. (**C**): The activity of CAT. Mean ± standard deviation (*n* = 3). Different numbers of symbols between treatments indicate significant differences.

In a comprehensive evaluation of the physiological and biochemical characteristics of the three samples, MeSY, MeZH, and MeRP, we measured the total antioxidant capacity (T-AOC), superoxide dismutase (SOD) activity, and catalase (CAT) activity (Figure 3). The results showed that the average T-AOC values across the three groups were highly consistent, ranging narrowly between 2.68 and 2.69 μmol/g. Similarly, SOD activity did not exhibit significant differences among the samples, with average values ranging from 53.96 to 54.90 U/g. Regarding CAT activity, although the average value for the MeRP sample (812.37 μmol/(min·g)) was slightly higher than that of the MeSY (797.44 μmol/(min·g)) and MeZH (798.51 μmol/(min·g)) samples, a statistical analysis confirmed that these differences were not statistically significant across the physiological and biochemical indicators examined (*p* > 0.05). In summary, the antioxidant indicators measured in this study were similar across different samples, with no significant differences observed.

### 3.5. Genetic Diversity

In a detailed statistical analysis of the genetic diversity parameters of the three populations, MeSY, MeZH, and MeRP, we observed that the MeSY population exhibited the highest SNP density, recorded at 5.39 SNP/Kb, compared to the MeRP population, which showed a lower SNP density of 2.62 SNP/Kb. The analysis of nucleotide diversity (π) also revealed higher genetic diversity in the MeSY population, with an average diversity value of 8.70 × 10⁻⁴. Regarding polymorphic information content (PIC), although the overall level was not high, the MeRP population had the highest PIC value at 0.15. Observations of heterozygosity (Ho) indicated that the MeRP population had the highest heterozygosity at 0.16, which may be related to its relatively high level of genetic diversity. In the analysis of the inbreeding coefficient (F_HOM_), the MeZH population had the highest value (Table 5). The calculation of the genetic differentiation index (F_ST_) between populations showed that the genetic differentiation between the MeSY and MeZH populations was the most significant (F_ST_ = 0.007), followed by that between the MeSY and MeRP populations with F_ST_ = 0.003, while the genetic differentiation between the MeZH and MeRP populations was the lowest (F_ST_ = 0.002).

## 4. Discussion

### 4.1. Analysis of Basic Nutritional Components in Different Populations of M. ensis

In the analysis of basic nutritional components in the three populations of *M. ensis*, the results showed no significant differences in ash content and crude protein levels (*p* > 0.05), with values ranging from 1.40 to 1.47 g/100 g for ash content, 74.50–75.30 g/100 g for moisture content and 20.80–22.10 g/100 g for crude protein. Compared to other shrimp species, the three *M. ensis* populations had lower ash content than the *Penaeus monodon* (1.46 g/100 g), *Litopenaeus vannamei* (1.52 g/100 g), and *Fenneropenaeus chinensis* (1.69 g/100 g) [25]. The crude protein content was higher (20.08–22.10 g/100 g) compared to *L. vannamei* (15.09 g/100 g), *Marsupenaeus japonicus* (14.60 g/100 g), *F. chinensis* (13.18 g/100 g), *P. monodon* (13.29 g/100 g), and *Procambarus clarkii* (12.33 g/100 g) [26]. The high crude protein content in shrimp muscle is one of the reasons for their classification as high-quality seafood. Additionally, the crude fat content in *M. ensis* (0.83 to 1.03 g/100 g) was lower compared to *M. japonicus* (2.09 g/100 g) [27] and similar to that of *L. vannamei* (0.8–1.1%) [28]. Overall, the three *M. ensis* populations have lower ash and crude fat content, and higher crude protein content, indicating that they have a higher protein nutritional value and lower caloric content, making them a high-protein, low-fat food option. Among the three populations, MeZH exhibited the highest levels of ash, moisture, and total sugar, while crude fat was highest in the MeRP population, and crude protein was most elevated in the MeSY population. Overall, MeZH consistently showed higher values across most parameters, placing it in a leading position among the three populations. Therefore, in terms of conventional nutritional components, MeZH stands out with superior nutritional values compared to the other two groups.

### 4.2. Analysis of Amino Acid Content in Different Populations of M. ensis

Amino acids play a crucial role in maintaining life functions. They are not only the fundamental units of proteins but also participate in regulating gene expression, enhancing antioxidant defenses, and modulating nitric oxide production, among other physiological processes [29]. The more abundant and diverse the amino acid content, the higher the nutritional value of the muscle [30]. In the three populations studied, the EAA content was moderate, ranging from 4.72 to 5.72 g/100 g, which is lower than that of *P. monodon* (5.44–6.41 g/100 g) [4] and *M. japonicus* (6.59 g/100 g) [31]. According to the ideal model proposed by FAO/WHO, high-quality proteins should have an EAA/TAA ratio of 40%. The three populations studied largely meet this criterion, indicating a good balance of amino acids and categorizing them as high-quality proteins for human consumption. Additionally, the palatability of animal proteins depends on the composition and content of DAA [32]. In this study, the MeSY population showed relatively high levels of SEAA and NEAA, with a particularly notable advantage in EAA and TAA levels. The EAA/TAA ratio approached the ideal 40%. Additionally, the DAA content also peaked at high levels. In contrast, the amino acid levels in the MeZH samples were generally lower than those in the MeSY population but higher than the MeRP population for certain amino acids. Overall, *M. ensis* exhibited high nutritional value and good palatability, with the MeSY population standing out for its highest amino acid content and superior performance.

### 4.3. Analysis of Fatty Acid Composition in Different Populations of M. ensis

The composition of fatty acids in muscle is another key factor influencing quality and flavor [33]. Monounsaturated fatty acids (MUFA) positively impact blood circulation, regulate blood sugar and lipid metabolism, and effectively lower cholesterol levels [34]. Additionally, MUFAs are essential for reproduction and growth, enhance the flavor of food when heated, reduce blood viscosity, and boost immune function [35]. Polyunsaturated fatty acids (PUFA), especially eicosapentaenoic acid (EPA) and docosahexaenoic acid (DHA), serve as major precursors for synthesizing bioactive anti-inflammatory mediators. They play a crucial role in brain development and in preventing inflammation associated with cardiovascular diseases [36,37].

In this study, the three populations of *M. ensis* all showed a trend of ΣPUFA > ΣMUFA. Specifically, ΣMUFA ranged from 75.1 to 104.2 mg/100 g, while ΣPUFA ranged from 181.15 to 221.20 mg/100 g, indicating higher values compared to *L. vannamei* (14.79–17.67 mg/100 g for ΣMUFA and 38.11–44.88 mg/100 g for ΣPUFA) [28] and *F. chinensis* (18.85 mg/100 g for ΣMUFA and 42.58 mg/100 g for ΣPUFA) [25]. Moreover, the DHA + EPA content in this study ranged from 133.15 to 161.55 mg/100 g, which is higher than that in *L. vannamei* (9.72 mg/100 g), *P. clarkii* (15.76 mg/100 g), *P. monodon* (16.29 mg/100 g), *F. chinensis* (7.98 mg/100 g), and *M. japonicus* (11.58 mg/100 g) [26]. This indicates that *M. ensis* has a high fatty acid content, reflecting its nutritional value and potential health benefits. In the three populations, MeRP demonstrated a leading position in all measured fatty acid types except for C20:2 and C22:1n9, indicating that the MeRP population has a richer fatty acid profile. The levels of C20:2 and C22:1n9 were higher in the MeZH population, while other fatty acids showed little difference between the MeSY and MeZH populations. Overall, MeRP stands out with significantly higher fatty acid content among the three populations, suggesting that it has greater nutritional value.

### 4.4. Analysis of Antioxidant Capacity in Different Populations of M. ensis

For aquatic organisms, the internal antioxidant mechanisms are crucial for survival and for defending against environmental stresses such as pollution, disease, temperature fluctuations, and changes in oxygen levels. These stress factors can exacerbate the production of reactive oxygen species (ROS). Aquatic organisms rely on an antioxidant enzyme system, including SOD and CAT, to neutralize these potentially harmful ROS, thereby maintaining cellular integrity and physiological function [38]. SOD is a vital antioxidant enzyme that eliminates superoxide anion radicals, protecting the organism from oxidative damage [39]. CAT effectively removes hydrogen peroxide by decomposing it into water and oxygen, helping to protect cells from oxidative damage [40]. T-AOC is an indicator of the overall antioxidant activity of all antioxidant substances in a biological sample, including both enzymes and small molecular antioxidants. It reflects the organism’s ability to clear excess ROS and is an important indicator for assessing an individual’s ability to counteract oxidative stress and prevent oxidative damage. High T-AOC values generally indicate a strong antioxidant defense system, which helps protect cells from oxidative damage and maintain health [41]. Therefore, higher T-AOC, SOD, and CAT levels indicate stronger antioxidant and immune capabilities. In this study, the T-AOC activity of MeSY, MeZH, and MeRP was 2.68–2.69 μmol/g, higher than that of *P. clarkii* (2.57 U/mg) [42], SOD activity was 53.96–54.90 U/g, higher than that of *P. monodon* (39.65–47.29 U/g) [4]. These results indicate that all three *M. ensis* populations exhibit high antioxidant enzyme activity, endowing them with significant antioxidant stress capabilities and better resistance to adverse conditions, making them a promising breed with potential for breeding programs. Upon comparing the three populations, it was discovered that the activities of the three types of antioxidant enzymes all exhibited a high degree of consistency. Although the CAT activity in the MeRP samples was slightly higher than in the MeSY and MeZH, statistical analysis indicated that these differences were not significant in terms of physiological and biochemical indicators (*p* > 0.05). Overall, the antioxidant indices of the three populations were similar, with no significant differences observed, suggesting that these groups have a consistent antioxidant defense mechanism.

### 4.5. Analysis of Genetic Diversity in Different Populations of M. ensis

Genetic diversity is the foundation of species evolution, and higher genetic diversity enhances a species’ ability to adapt to environmental changes, grow, and resist diseases [43]. Currently, there is limited research on the genetic diversity of *M. ensis*. Nguyen Xuan Huy et al. [11] first observed the genetic diversity of four populations of *M. ensis* from the Mekong Delta-Khu Lake in Vietnam, revealing a high level of genetic variation with most genetic differences occurring within populations, indicating a high level of gene flow. In this study, we analyzed genetic diversity parameters, including SNP density, nucleotide diversity, polymorphic information content, observed heterozygosity, inbreeding coefficient, and genetic differentiation index. SNP density reflects the frequency of SNP sites in the genome, with high SNP density typically associated with greater genetic variability [44]. Nucleotide diversity reflects the degree of variation at nucleotide sites within a gene locus and is an important indicator of population genetic diversity [45]. The F_ST_ value is used to assess the degree of genetic differentiation between populations, with high F_ST_ values indicating significant genetic isolation [46].

In our analysis of the MeSY, MeZH, and MeRP populations, we found that SNP density ranged from 2.62 to 5.39, nucleotide diversity (π) ranged from 5.34 × 10−^4^ to 8.70 × 10^−4^, and observed heterozygosity (Ho) ranged from 0.07 to 0.16. These indicators all showed relatively low genetic diversity. Additionally, the inbreeding coefficient (F_ST_) for these three populations ranged from 0.002 to 0.007, also indicating low values. These results suggest that the genetic variability among the three *M. ensis* populations is low, with no significant population differentiation. Among them, the MeSY population exhibited relatively higher SNP density and nucleotide diversity and had a higher inbreeding coefficient compared to the MeZH population. This suggests that within these three populations, the MeSY group has relatively higher genetic diversity and shows a relatively higher degree of genetic differentiation compared to the MeZH group.

## 5. Conclusions

This study provides an in-depth analysis of the germplasm resources of three different populations of *M. ensis*. The results indicate that these prawns possess a rich array of nutritional components, including various amino acids and fatty acids, and exhibit strong antioxidant capabilities. These attributes give *M. ensis* significant advantages in terms of nutritional value and health benefits. However, the analysis also reveals that the genetic diversity within these populations is relatively low, and there is minimal genetic differentiation among them. This finding highlights the limited genetic variation currently present among these populations but also provides direction for future germplasm improvement and management.

This study provides compelling evidence demonstrating the advantages of germplasm resources from different regions through a comprehensive evaluation of conventional nutritional components, amino acids, fatty acids, antioxidant capacity, and genetic diversity. The findings offer valuable insights into the germplasm characteristics and breeding potential of *M. ensis*. Notably, *M. ensis* exhibits not only a superior nutritional composition and antioxidant capacity, but also low genetic differentiation despite its rich nutritional profile, positioning it as an ideal candidate for widespread aquaculture development. Future efforts in genetic improvement and selective breeding could further enhance its genetic diversity, thereby increasing both its adaptability and production potential.

## Figures and Tables

**Table 1:** Basic information of the survey point.

Population	Body Length (mm)	Weight (g)	Location
Sanya (MeSY)	112.00 ± 12.00	29.60 ± 17.82	18°30′ N, 110°10′ E
Zhuhai (MeZH)	124.00 ± 18.00	18.95 ± 13.75	22°14′ N, 113°36′ E
Raoping (MeRP)	121.00 ± 18.00	19.20 ± 8.90	23°66′ N, 117°00′ E

Body length and weight values are mean ± standard deviation (*n* = 30).

**Table 2:** Muscle general nutrition composition and content of three groups of *M. ensis*.

Nutritional Components	MeSY	MeZH	MeRP
Ash (g/100 g)	1.40 ± 0.10 ^a^	1.47 ± 0.07 ^a^	1.40 ± 0.00 ^a^
Moisture (g/100 g)	74.50 ± 0.90 ^a^	75.30 ± 1.20 ^a^	74.90 ± 0.50 ^a^
Crude fat (g/100 g)	0.97 ± 0.13 ^a^	0.83 ± 0.07 ^b^	1.03 ± 0.07 ^a^
Crude protein (g/100 g)	22.10 ± 0.80 ^a^	20.80 ± 0.60 ^a^	21.17 ± 0.17 ^a^
Total sugar (%)	0.34 ± 0.03 ^a^	0.36 ± 0.03 ^a^	0.29 ± 0.04 ^b^

Mean ± standard deviation (*n* = 3). Superscript letters (a, b) within the same row denote statistically significant differences between groups. Values sharing the same letter are not significantly different (*p* > 0.05).

**Table 3:** Muscle amino acid composition and content of three groups of *M. ensis*.

Amino Acid (g/100 g)	MeSY	MeZH	MeRP
Aspartic acid ^@^	1.59 ± 0.02 ^a^	1.37 ± 0.03 ^b^	1.53 ± 0.01 ^a^
Threonine *	0.61 ± 0.0 ^a^	0.54 ± 0.02 ^b^	0.59 ± 0.01 ^a^
Serine	0.50 ± 0.0 ^a^	0.49 ± 0.01 ^a^	0.54 ± 0.01 ^a^
Glutamic acid ^@^	2.31 ± 0.03 ^a^	2.02 ± 0.04 ^b^	2.26 ± 0.01 ^a^
Glycine ^@^	1.56 ± 0.02 ^a^	1.35 ± 0.03 ^b^	1.42 ± 0.02 ^b^
Alanine ^@^	0.94 ± 0.02 ^a^	0.84 ± 0.02 ^b^	0.90 ± 0.01 ^a^
Cystine	0.15 ± 0.00 ^a^	0.13 ± 0.00 ^a^	0.09 ± 0.01 ^b^
Valine *	0.72 ± 0.01 ^a^	0.59 ± 0.01 ^b^	0.55 ± 0.01 ^b^
Methionine *	0.34 ± 0.01 ^a^	0.30 ± 0.01 ^a^	0.31 ± 0.03 ^a^
Isoleucine *	0.66 ± 0.01 ^a^	0.53 ± 0.01 ^b^	0.48 ± 0.01 ^b^
Leucine *	1.26 ± 0.02 ^a^	1.06 ± 0.02 ^b^	1.16 ± 0.02 ^c^
Tyrosine	0.33 ± 0.0 ^a^	0.34 ± 0.01 ^a^	0.44 ± 0.01 ^b^
Phenylalanine *	0.64 ± 0.00 ^a^	0.54 ± 0.01 ^b^	0.58 ± 0.02 ^b^
Lysine *	1.49 ± 0.02 ^a^	1.16 ± 0.02 ^b^	1.26 ± 0.02 ^c^
Histidine ^&^	0.30 ± 0.00 ^a^	0.25 ± 0.01 ^a^	0.27 ± 0.01 ^a^
Arginine ^&^	1.64 ± 0.02 ^a^	1.47 ± 0.03 ^b^	1.59 ± 0.04 ^a^
Proline	0.68 ± 0.00 ^a^	0.55 ± 0.01 ^b^	0.67 ± 0.01 ^a^

Mean amino acids ± standard deviation (*n* = 3). Different letters between treatments indicate significant differences (*p* < 0.05). ^@^ Delicious amino acids. * Essential amino acids. ^&^ Semi-essential amino acids. Superscript letters (a–c) within the same row denote statistically significant differences between groups. Values sharing the same letter are not significantly different (*p* > 0.05).

**Table 4:** Muscle fatty acid composition and content of three groups of *M. ensis*.

Fatty Acid(mg/100 g)	MeSY	MeZH	MeRP
C16:0	68.40 ± 4.80 ^a^	62.00 ± 3.20 ^b^	89.00 ± 3.70 ^c^
C16:1	16.40 ± 1.20 ^a^	14.50 ± 0.70 ^a^	23.90 ± 1.00 ^b^
C17:0	13.30 ± 0.90 ^a^	12.30 ± 0.70 ^a^	16.80 ± 0.70 ^b^
C18:0	79.30 ± 5.00 ^a^	75.40 ± 3.70 ^b^	94.10 ± 3.80 ^c^
C18:1n9c	45.40 ± 3.20 ^a^	53.00 ± 3.20 ^b^	63.50 ± 2.60 ^c^
C18:2n6c	6.00 ± 0.40 ^a^	5.20 ± 0.20 ^a^	8.70 ± 0.40 ^b^
C20:2	4.40 ± 0.40 ^a^	5.45 ± 0.25 ^a^	4.65 ± 0.25 ^a^
C22:0	9.55 ± 0.75 ^a^	9.60 ± 0.40 ^a^	11.05 ± 0.45 ^b^
C20:4n6	37.60 ± 2.60 ^a^	37.35 ± 1.35 ^a^	46.30 ± 1.90 ^b^
C22:1n9	13.95 ± 0.85 ^a^	25.75 ± 0.85 ^b^	13.85 ± 0.55 ^a^
C20:5n3(EPA)	68.60 ± 4.90 ^a^	69.50 ± 3.50 ^a^	82.40 ± 3.50 ^b^
C22:6n3(DHA)	64.55 ± 4.35 ^a^	66.10 ± 3.30 ^a^	79.15 ± 2.85 ^b^

Mean fatty acids ± standard deviation (*n* = 3). Different letters between treatments indicate significant differences (*p* < 0.05). C16:0—Palmitic acid. C16:1—Palmitoleic acid. C17:0—Heptadecanoic acid. C18:0—Stearic acid. C18:1n9c—Oleic acid. C18:2n6c—Linoleic acid. C20:2—Eicosadienoic acid. C22:0—Behenic acid. C20:4n6—Arachidonic acid. C22:1n9—Erucic acid. C20:5n3 (EPA)—Eicosapentaenoic acid. C22:6n3 (DHA)—Docosahexaenoic acid. Superscript letters (a–c) within the same row denote statistically significant differences between groups. Values sharing the same letter are not significantly different (*p* > 0.05).

**Table 5:** Statistics of genetic diversity parameters of three populations of *M. ensis.*

Population	MeSY	MeZH	MeRP
SNP density (SNP/Kb)	5.39	2.79	2.62
Nucleotide diversity (π)	8.70 × 10^−4^ ± 1.16 × 10^−3^	5.34 × 10^−4^ ± 7.59 × 10^−4^	5.52 × 10 ^−4^ ± 7.79 × 10^−4^
Polymorphism information content (PIC)	1.23 × 10^−1^ ± 8.44 × 10^−2^	0.14 ± 0.11	0.15 ± 0.11
Observed heterozygosity (Ho)	6.99 × 10^−2^ ± 1.30 × 10^−1^	0.14 ± 0.16	0.16 ± 0.17
Inbreeding coefficient (F_HOM_)	4.83 × 10^−1^ ± 6.09 × 10^−2^	8.31 × 10^−2^ ± 6.97 × 10 ^−2^	5.93 × 10^−2^ ± 2.48 × 10^−2^

## Data Availability

No new data were created or analyzed in this study.
